# Intersecting Burdens: Pain and Psychosocial Stressors in Women with HIV and Type 2 Diabetes – Insights from the MACS/WIHS Combined Cohort Study

**DOI:** 10.21203/rs.3.rs-8865542/v1

**Published:** 2026-04-14

**Authors:** Kristen Allen-Watts, Jessica Blair, Zenoria Causey-Pruitt, Nicole Beaulieu Perez, Clementine Atkinson, Georgiana Logan, Lauren Frances Collins, Burel Goodin, Andrea Cherrington, Jessica Jaiswal, Taylor L Taylor, Raymond Jones, Emily B Levitan, Mirjam-Colette Kempf

**Affiliations:** University of Alabama at Birmingham; University of Alabama at Birmingham; University of Alabama at Birmingham; New York University; University of Alabama at Birmingham; Marshall University; Emory University; Washington University in St. Louis; University of Alabama at Birmingham; University of Alabama at Birmingham; University of Alabama at Birmingham; University of Alabama at Birmingham; University of Alabama at Birmingham; University of Alabama at Birmingham

**Keywords:** HIV, diabetes, pain, mental health

## Abstract

**Introduction:**

HIV has transitioned into a chronic condition often accompanied by multimorbidity, including type 2 diabetes (T2D). Women with HIV (WWH) and T2D face elevated risks for depression, loneliness, and reduced social support. Pain—a common and burdensome symptom in both conditions—remains underexamined despite its potential to exacerbate psychosocial challenges and diminish quality of life.

**Methods:**

We conducted a cross-sectional analysis using data from the MACS/WIHS Combined Cohort Study (MWCCS), focusing on the Women’s Interagency HIV Study (WIHS) subset. Self-reported measures included pain status (dichotomized as yes/no) and mental health outcomes (depressive symptoms, loneliness, social support, and quality of life). Clinical variables included HIV serostatus, diabetes status, hemoglobin A1c, and body mass index. Generalized linear regression models assessed associations between pain and mental health outcomes, adjusting for demographic and clinical covariates.

**Results:**

Among 2,410 participants, 72% identified as Non-Hispanic Black, 10% as Non-Hispanic White, 15% as Hispanic, and 4% as other; mean age was 47.4 years (SD = 9.3) and BMI 31.8 (SD 8.9). Seventy-one percent were HIV seropositive, 19% had diabetes, and 67% reported pain. Overall, participants reported moderate social support (mean = 57.1, range 15–75), with higher scores among women with diabetes (mean = 66.3) compared to those without diabetes (mean = 57.3). HIV status did not significantly influence social support. Fully adjusted models revealed strong associations between pain and all mental health outcomes (p < 0.001). Women reporting pain had higher depressive symptoms (B = 7.59; 95% CI: 6.68, 8.49), greater loneliness (B = 0.80; 95% CI: 0.65, 0.94), lower social support (B = − 5.37; 95% CI: −6.64, − 4.10), and markedly lower quality of life (B = − 23.44; 95% CI: −24.87, − 22.01) compared to women without pain. No significant interactions were observed by HIV or diabetes status.

**Conclusions:**

Pain is strongly associated with worse psychosocial outcomes among women in WIHS, regardless of HIV or diabetes status. These findings highlight the need for integrated, patient-centered interventions that address pain alongside mental health and social support to improve quality of life for women with multimorbidity. Future research should explore longitudinal patterns and tailored strategies to mitigate the compounded burden of pain and psychosocial distress.

## BACKGROUND

The advent of combination antiretroviral therapy (ART), has transformed HIV from a fatal infection into a chronic condition, significantly extending life expectancy for people with HIV (PWH) [1, 2]. As survival improves, PWH increasingly experience multimorbidity [3] including non-HIV-related conditions such as type 2 diabetes (T2D), cardiovascular disease, and chronic pain [4–6] These comorbidities often intersect with psychosocial challenges, creating a complex health landscape that disproportionately affects women with HIV (WWH) [7].

Mental health concerns, particularly depression, are highly prevalent among WWH, with rates exceeding those of women without HIV and men with HIV [8]. Depression is associated with poorer quality of life, reduced treatment adherence, and adverse health outcomes [9–12]. Similarly, women with T2D face elevated depression risk, driven by disease management demands and complications [9, 10]. Both conditions have also been linked to diminished psychosocial well-being [13]. Loneliness and low social support further compound these challenges, as they are associated with stigma, isolation, and poorer self-management in HIV and T2D [14–20]. Social support, conversely, is a protective factor that can buffer these stressors, though barriers such as fear of disclosure and discrimination often limit access [21–23].

Pain is another common and burdensome symptom in both HIV [5, 24, 25] and T2D [26, 27]. Beyond its physical impact, pain contributes to psychological distress and impaired social functioning [28–30]. Despite its prevalence, pain remains understudied in the context of co-occurring HIV and T2D, particularly among women. Understanding how pain relates to mental health outcomes, such as depressive symptoms, loneliness, social support, and quality of life, and whether these associations differ by HIV and diabetes status is critical for informing interventions that address the complex needs of women living with diabetes and HIV.

Therefore, this study aimed to examine the association between pain and mental health outcomes among women enrolled in the Women’s Interagency HIV Study (WIHS). We also assessed whether these associations vary by HIV serostatus and diabetes status, given the potential for compounded psychosocial burden in these subgroups.

## METHODS

We conducted a cross-sectional analysis from the MACS/WIHS Combined Cohort Study (MWCCS), focusing on the WIHS subset, to examine the association between pain and mental health outcomes at the end of observation, as well as the potential modifying effects of HIV serostatus and diabetes. WIHS is a multicenter, prospective cohort study initiated in 1993 to explore the natural history and related comorbidities of HIV among women living with or at risk for HIV [31]. Details of the full protocol have been described in detail elsewhere [32–35]. Briefly, WIHS collected data biannually at sites across the U.S., including, Atlanta, GA; Birmingham, AL/Jackson, MS; Chapel Hill, NC; Chicago, IL; Miami, FL; Bronx, NY; Brooklyn, NY; Los Angeles, CA; San Francisco, CA; and Washington, DC until 2018. In 2019, WIHS merged with the Multicenter AIDS Cohort Study (MACS)—a parallel study of men—to form MWCCS. For this analysis, only WIHS participants were included.

### Study Population

For the cross-sectional analysis, we included WIHS participants with complete visit data from 2013–2019. Participants were excluded if they were missing the pain questionnaire or mental health outcomes (i.e., depressive symptoms, loneliness, social support, and quality of life).

All study procedures were reviewed and approved by the appropriate institutional review boards. This analysis received approval by the MWCCS Executive Committee. Data necessary to replicate these analyses are available from MWCCS [36]. Participants provided written informed consents prior to data collection.

#### Main Exposure

Pain was assessed by a psychosocial measure questionnaire utilizing two Likert scale components. The first component ascertained the amount of bodily pain within the past 4 weeks: none, very mild, mild, moderate, severe, and very severe. The second component asked if bodily pain interfered with normal work within the past 4 weeks: not at all, slightly, moderately, quite a bit, or extremely. Pain was dichotomized as yes/no based-on participants reporting very mild to very severe bodily pain or reporting slightly to extreme interference with work.

#### Clinical Characteristics

Consistent with prior work in this cohort, diabetes was defined as meeting any of the following criteria: self-reported use of anti-diabetic medications, fasting glucose ≥ 126 mg/dL, hemoglobin A1c (HbA1c) ≥ 6.5%, or a confirmed self-reported diagnosis of diabetes. HIV serostatus was defined as women with or without HIV, which was assessed using ELISA with western blot confirmation.

#### Mental Health Outcomes

Depressive symptoms were measured using the Center for Epidemiologic Studies Depression Scale (CES-D). This 20-item measure has a scoring range of 0–60, with scores of 16 or more signaling likely depression [37]. Scores are calculated by asking respondents to rate the frequency at which they have experienced depression-associated symptoms in the past week, with 0 being rarely or never, and 3 being always or almost always [37].

Loneliness was measured using the 3-item Loneliness scale. Respondents rate their frequency loneliness and isolation on a scale from 1 to 3; 1 being hardly ever, 2 being some of the time, and 3 being often. Scores can range from 3–9, with higher scores indicating greater loneliness [38].

Social support was measured on a scale from 1 to 5, with scores ranging from 15 to 75. Respondents rated their feelings and experiences of social support, (i.e. being listened to, feeling understood and supported, receiving advice, assistance preparing meals or getting to doctors’ appointments, etc.) from 1 = none, 2 = a little, 3 = some, 4 = most, 5 = all the time [39, 40].

Quality of life was measured using the Quality-of-life Index variable from sociodemographic summary WIHS dataset. Quality of life was measured using the QLINDX variable from the SOCDEM summary dataset of the Women’s Interagency HIV Study (WIHS), a weighted composite index incorporating physical, emotional, social, and role functioning domains [33, 41–43].

#### Covariates

We included baseline self-reported demographics on race and ethnicity and the highest level of education (less than high school, high school graduate, some college, and college graduate). Participants were of Hispanic origin if they answered ‘yes’ to the question “Are you of Hispanic or Latin origin?” Race was self-reported as White, Black, Asian, Pacific Islander, American Indian/Alaska Native, and other. Due to limited sample size of some groups, we categorized race/ethnicity as Hispanic (of any race), non-Hispanic Black, non-Hispanic White, and non-Hispanic Other.

The following variables were reported at the time of visit: age (years), body mass index (kg/m^2^), average annual household income >$12,000, employment status (yes/no), and health insurance, which includes Ryan White or AIDS Drug Assistance Programs. Marital status was categorized as married (including legal/common law married, and not married but living with partner), divorced (including annulled/separated), widowed, never married, and other. In addition, the South was categorized as sites located in Chapel Hill, NC; Atlanta, GA; Miami, FL; and Birmingham, AL/Jackson, MS.

### Statistical Analysis

Characteristics were shown for the study sample and stratified by diabetes status and HIV serostatus. Continuous variables show the mean (standard deviation) while categorical variables show counts (frequencies). Statistical significance was set at α < 0.05 using pooled equality of variance for continuous variables and chi-square test for categorical variables.

We utilized generalized linear regression for continuous outcomes to test the association between the main exposure of pain and mental health outcomes (i.e., depressive symptoms, loneliness, social support, and quality of life), adjusting for covariates selected based on their established relationship to mental health outcomes [44]. The crude model included pain status only. Model 1 included additional variables of age and race/ethnicity. Model 2 included additional variables of income, health insurance, employment, education, marital status, and region. Model 3, which is the fully adjusted model, included additional variables of HIV serostatus, diabetes status, and body mass index. Statistical significance was set at α < 0.05 using type 3 sum of squares. The normality of residuals for each outcome was assessed using normal Q-Q plots. Additionally, we tested the interaction of age, diabetes, and HIV serostatus with exposure of pain for each mental health outcome in fully adjusted models. Stratifications were conducted if interactions were significant at α < 0.05.

Multiple imputation using the fully conditional specification method, a seed for reproducibility, and 10 imputations for each missing covariate was used for the following: income, health insurance, employment status, education, marital status, and body mass index. These variables were considered to be missing at random. All analyses were conducted using SAS version 9.4 (SAS Institute).

## RESULTS

**Figure 1.**
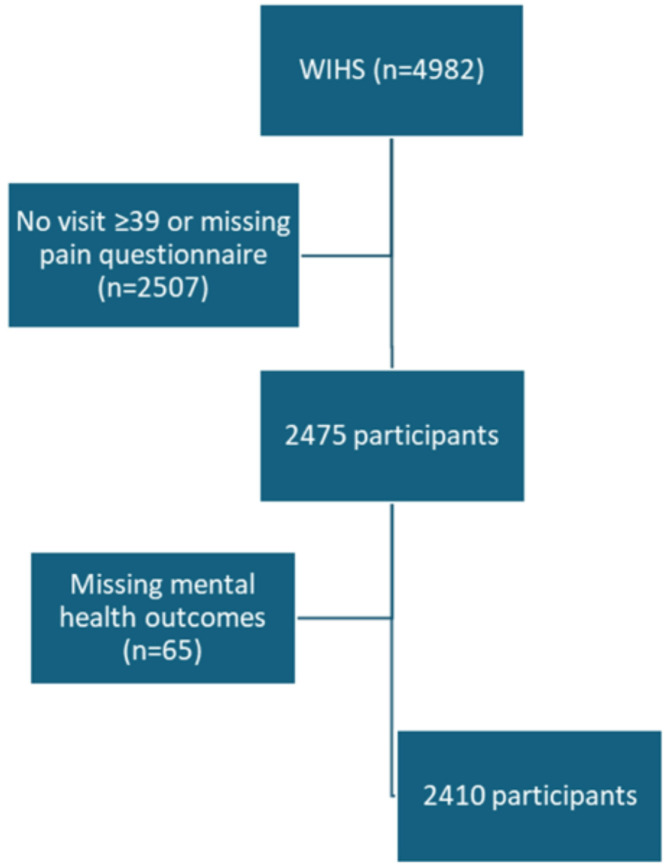
Flowchart Diagram of Exclusion Criteria for Study Sample

The participant flow diagram is shown in Fig. 1 with exclusion criteria, and participant characteristics for women included in our sample are displayed in Table 1. Of the 2410 women, 72% were Non-Hispanic Black (NHB), 10% were Non-Hispanic White (NHW), 15% were Hispanic, and 4% identified as other. The mean age was 47.4 years (SD 9.3). Forty-nine percent had an annual average income greater than $12,000, 91% had insurance, and 35% were currently employed. One third of the sample had less than a HS education and 33% were never married. The mean BMI was 31.8 (SD 8.9), 33% lived in the South and 71% were HIV seropositive. Nineteen percent had diabetes and 67% reported experiencing pain.

Among women with diabetes (n = 448), the mean age of participants was 51.4 (SD 8.1), 69% were NHB 42% had an income greater than $12,000, 94% had insurance, and 23% were employed. Thirty-seven percent had less than a HS education, 29% were married, 24% lived in the South, and 68% were HIV seropositive. The mean BMI was 35 (SD 9.1) and 79% reported experiencing pain.

Among women with HIV (1698), the mean age was 47.9 (SD 9.1), 72% were NHB, 48% had an income greater than $12,000, 96% had insurance, and 32% were employed. Thirty-three percent had less than a HS education, 33% had never been married, 34% lived in the South, and 67% reported experiencing pain. The mean BMI was 31.4 (SD 9.0).

Overall, the mean score for participants’ CES-D score was 12.2 (± 11.5). For loneliness, the mean score was 4.4 (± 1.7), social support, 57.1 (± 15.0), and quality of life 70.3 (± 21.0). The mean social support scores for those with or without diabetes were 66.3 (± 14.7) and 57.3 (± 15.1), respectively. The mean social support score for women with or without HIV were 56.9 (± 15.1) and 57.6 (± 14.8), respectively.

Linear regressions showing the relationship between pain versus no pain and mental health outcomes are displayed in Table 2. We tested the fully adjusted models for each outcome with interactions of pain and age, pain and diabetes, and pain and HIV serostatus. We did not find any significant interactions among any of the main outcomes of mental health. All mental health outcomes passed normality. Our results suggest that participants reporting pain had a 7.59 higher average depressive symptoms score (B = 7.59; CI: 6.68,8.49; p = < 0.001) compared to participants without pain. Similarly, participants reporting pain had a 0.80 higher average loneliness score (B = 0.80; CI: 0.65, 0.94; p = < 0.001) compared to participants without pain. For social support, participants reporting pain had a 5.37 lower average social support score compared to participants without pain (B= −5.37; CI: −6.64, −4.10; p = < 0.001). Regarding QOL, participants reporting pain had a 23.44 lower average QOL score (B= −23.44; CI: −24.87, −22.01; p = < 0.001) compared to participants without pain.

## Discussion

This study examined the relationship between pain and mental health outcomes among women enrolled in the WIHS cohort, with attention to differences by HIV serostatus and diabetes status. While prior research often reports elevated rates of depression and loneliness and low social support among PWH [17, 45–47] and T2D [48], our findings diverge from this narrative. Participants reported relatively low levels of depressive symptoms and loneliness, and moderate levels of perceived social support.

One possible explanation for these findings is the unique context of WIHS. Long-term engagement in a structured cohort may foster trust, continuity of care, and access to resources that buffer psychosocial distress. Overall, participants reported moderate social support (mean score 57.1 on a scale of 15–75), but subgroup differences were notable. Women with diabetes had substantially higher social support scores (mean 66.3) compared to those without diabetes (mean 57.3), suggesting that diabetes may be associated with stronger support networks, possibly due to increased healthcare engagement or reliance on caregiving resources. In contrast, social support scores were similar for WWH (mean 56.9) and those without HIV (mean 57.6), indicating that HIV status did not significantly influence perceived support in this cohort. Prior studies have shown that perceived social support mitigates stigma and improves mental health outcomes in WWH [49] and plays a protective role in diabetes management [50]. WIHS participants may benefit from both formal and informal support networks, which could explain these patterns compared to population-based studies.

Despite these strengths, pain was strongly associated with worse psychosocial outcomes. Women reporting pain had significantly higher depressive symptoms and loneliness and lower social support and quality of life compared to those without pain. Quality of life showed the largest inverse association with pain, underscoring its importance as a target for intervention. These findings align with prior research linking chronic pain to emotional distress and impaired functioning [51]. Neurobiological mechanisms may contribute, as chronic pain is associated with dysregulation of the hypothalamic-pituitary-adrenal axis and heightened activity in brain regions involved in emotional processing, which can amplify distress and reduce coping capacity.

Subgroup analyses did not reveal significant interactions by HIV or diabetes status, suggesting that the negative impact of pain on mental health is pervasive across these groups. However, descriptive differences, such as higher pain prevalence among women with diabetes, highlight the need for integrated approaches that address both physical and psychosocial dimensions of health in women with multimorbidity.

This study has several limitations that should be considered when interpreting the findings. First, the data were drawn from the WIHS cohort, a long-standing observational cohort with structured follow-up and research engagement, which may limit generalizability to broader populations of women with HIV and/or T2D. WIHS participants may have greater access to healthcare, research resources, and social support than women outside of the study, potentially influencing psychosocial outcomes [33]. Additionally, while the study design allows for robust cross-sectional associations, it does not permit causal inference.

Longitudinal data would be needed to determine the directionality of relationships between pain, emotional distress, social support, and quality of life. Future research should include longitudinal designs to assess how pain and psychosocial outcomes evolve over time, and whether targeted interventions—such as peer support or integrated behavioral health—can buffer these effects. Qualitative studies may also provide deeper insight into the lived experiences and coping strategies of women managing multimorbidity in resource-limited settings. Additionally, factors such as race, gender identity, socioeconomic status, and diabetes-related distress warrant further investigation as potential modifies of. Pain and mental health outcomes. Finally, reliance on self-reported measures introduces possibility of reporting bias.

## CONCLUSIONS

In conclusion, our findings demonstrate that pain is strongly associated with worse psychosocial outcomes among women in the WIHS cohort, including higher depressive symptoms and loneliness and lower social support and quality of life. While overall levels of depressive symptoms and loneliness were relatively low and social support was moderate, women with diabetes reported notably higher social support compared to those without diabetes, whereas HIV status did not significantly influence support levels. These results underscore the pervasive impact of pain across subgroups and highlight quality of life as the outcome most adversely affected. Interventions that integrate pain management with strategies to enhance psychosocial well-being, such as strengthening social support networks and addressing emotional distress, are essential for improving health outcomes in women with diabetes and HIV. Future research should explore longitudinal patterns and test tailored approaches that combine behavioral health, peer support, and chronic disease management to mitigate the compounded burden of pain and psychosocial stressors.

## Figures and Tables

**Table 1:** Sociodemographic Distributions of Women in the MACS/WIHS Combined Cohort, by Diabetes Status, and by HIV Serostatus

Variable	Overall (n = 2410)	Diabetes (n = 448)	No diabetes (n = 1962)	p-value	WWH (n = 1698)	WWOH (n = 712)	p-value
Age at visit, mean (SD)	47.4 ± 9.3	51.4 ± 8.1	46.5 ± 9.4	**< 0.001**	47.9 ± 9.1	46.3 ± 9.8	**< 0.001**
Race/ethnicity				0.09			**0.01**
NH Black	1738 (72.1)	310 (69.2)	1428 (72.8)		1223 (72.0)	515 (72.3)	
NH White	232 (9.6)	38 (8.5)	194 (9.9)		180 (10.6)	52 (7.3)	
Hispanic	353 (14.7)	81 (18.1)	272 (13.9)		243 (14.3)	110 (15.5)	
NH Other	87 (3.6)	19 (4.2)	68 (3.5)		52 (3.1)	35 (4.9)	
Income >$12,000	1128 (49.0)	179 (42.0)	949 (50.6)	**0.001**	773 (47.5)	355 (52.5)	**0.03**
Insurance	2192 (91.1)	419 (93.5)	1773 (91.0)	0.05	1622 (95.7)	570 (80.2)	**< 0.001**
Employed	840 (34.9)	105 (23.4)	735 (37.5)	**< 0.001**	545 (32.2)	295 (41.4)	**< 0.001**
Highest level of education				0.09			0.45
< HS	787 (32.7)	165 (36.8)	622 (31.7)		562 (33.1)	225 (31.7)	
HS graduate	761 (31.6)	141 (31.5)	620 (31.6)		546 (32.2)	215 (30.2)	
Some college	674 (28.0)	116 (25.9)	558 (28.5)		460 (27.1)	214 (30.1)	
≥ College graduate	186 (7.7)	26 (5.8)	160 (8.2)		129 (7.6)	57 (8.0)	
Marital status				**< 0.001**			0.05
Married	711 (30.5)	124 (28.8)	587 (30.8)		498 (30.2)	213 (31.1)	
Divorced	462 (19.8)	98 (22.7)	364 (19.1)		328 (19.9)	134 (19.5)	
Widowed	164 (7.0)	44 (10.2)	120 (6.3)		131 (7.9)	33 (4.8)	
Never married	763 (32.7)	108 (25.1)	655 (34.4)		537 (32.6)	226 (32.9)	
Other	235 (10.1)	57 (13.2)	178 (9.4)		155 (9.4)	80 (117)	
BMI (kg/m^2^), mean (SD)	31.8 ± 8.9	34.6 ± 9.1	31.2 ± 8.8	**< 0.001**	31.4 ± 9.0	32.8 ± 8.7	**< 0.001**
South	800 (33.2)	109 (24.3)	691 (35.2)	**< 0.001**	579 (34.1)	221 (31.0)	0.15
HIV seropositive	1698 (70.5)	304 (67.9)	1394 (71.1)	0.18	--	--	--
Diabetes	448 (18.6)	--	--	--	304 (17.9)	144 (20.2)	0.18
*Pain*	1609 (66.78)	353 (78.8)	1256 (64.0)	--	1138 (67.0)	471 (66.2)	--
*Depression*	12.2 ± 11.5	13.4 ± 11.5	11.9 ± 11.5	--	12.1 ± 11.3	12.4 ± 11.9	--
*Loneliness*	4.4 ± 1.7	4.4 ± 1.8	4.4 ± 1.7	--	4.4 ± 1.8	4.3 ± 1.7	--
*Social support*	57.1 ± 15.0	56.3 ± 14.7	57.3 ± 15.1	--	56.9 ± 15.1	57.6 ± 14.8	--
*QOL*	70.3 ± 21.0	65.5 ± 20.4	71.4 ± 21.0	--	70.1 ± 20.9	70.8 ± 21.1	--

SD, standard deviation, NH, non-Hispanic; HS, high school; QoL, quality of life, BMI, Body Mass Index.

**Table 2:** Linear Regression Models showing the Relationship Between Pain and Mental Health Outcomes

	Depressive symptoms		
(Pain vs no pain)	*Difference (SE)*	*95% CI*	*p-value*
Crude	8.06 (0.47)	(7.15, 8.98)	**< 0.001**
Model 1	8.22 (0.48)	(7.29, 9.16)	**< 0.001**
Model 2	7.46 (0.46)	(6.55, 8.36)	**< 0.001**
Model 3	7.59 (0.46)	(6.68, 8.49)	**< 0.001**
	Loneliness		
	*Difference (SE)*	*95% CI*	*p-value*
Crude	0.88 (0.07)	(0.74, 1.03)	**< 0.001**
Model 1	0.88 (0.07)	(0.74, 1.03)	**< 0.001**
Model 2	0.78 (0.07)	(0.64, 0.93)	**< 0.001**
Model 3	0.80 (0.07)	(0.65, 0.94)	**< 0.001**
	Social support		
	*Difference (SE)*	*95% CI*	*p-value*
Crude	−5.97 (0.64)	(−7.22, −4.72)	**< 0.001**
Model 1	−5.89 (0.65)	(−7.16, −4.62)	**< 0.001**
Model 2	−5.20 (0.65)	(−6.46, −3.93)	**< 0.001**
Model 3	−5.37 (0.65)	(−6.64, −4.10)	**< 0.001**
	Quality of life		
	*Difference (SE)*	*95% CI*	*p-value*
Crude	−25.55 (0.74)	(−27.00, −24.09)	**< 0.001**
Model 1	−24.75 (0.75)	(−26.23, −23.27)	**< 0.001**
Model 2	−23.56 (0.72)	(−24.97, −22.14)	**< 0.001**
Model 3	−23.44 (0.73)	(−24.87, −22.01)	**< 0.001**

Crude: pain status

Model 1: addition of age, race/ethnicity

Model 2: addition of income, insurance, employment, education, marital status, region

Model 3: addition of HIV serostatus, diabetes, body mass index (fully adjusted)

Type 3 sum of squares

## Data Availability

Access to individual-level data from the MACS/WIHS Combined Cohort Study Data (MWCCS) may be obtained upon review and approval of a MWCCS concept sheet. Data necessary to replicate these analyses are available from MWCCS ([mwccs@jhu.edu](mailto:mwccs@jhu.edu)).

## Abbreviations

ART: Antiretroviral therapy
HIV: Human immunodeficiency virus
PWH: People with HIV
T2D: Type 2 diabetes
WWH: Women with HIV
WIHS: Women’s Interagency HIV Study
MACS: Multicenter AIDS Cohort Study
MWCCS: WIHS Combined Cohort Study
HbA1c: Hemoglobin A1c
CES-D: Center for Epidemiologic Studies Depression Scale
NHB: Non-Hispanic Black
NHW: Non-Hispanic White
